# Research trends and emerging topics in gut microbiota-derived metabolites and diabetic kidney disease: a multi-database bibliometric analysis (2014–2025)

**DOI:** 10.3389/fmicb.2026.1967850

**Published:** 2026-10-09

**Authors:** Xinyu Yang, Xiangning Huang, Yaru Shi, Yunfeng Yu, Rong Yu

**Affiliations:** School of Traditional Chinese Medicine, Hunan University of Chinese Medicine, Changsha, Hunan, China

**Keywords:** bibliometrics, diabetic kidney disease, gut microbial metabolites, gut-kidney axis, visualization analysis

## Abstract

**Objective:**

Diabetic kidney disease (DKD) is a leading cause of end-stage renal disease. The gut-kidney axis has highlighted the potential involvement of gut microbial metabolites in the development and progression of DKD. This study aimed to systematically characterize the knowledge structure, research hotspots, collaboration networks, and emerging trends at the intersection of DKD and gut microbial metabolites from 2014 to 2025 using a multidatabase bibliometric approach.

**Methods:**

The Web of Science Core Collection, PubMed, Embase, and Scopus were systematically searched from database inception to December 31, 2025, using a combination of controlled vocabulary and free-text terms. Only original articles and reviews published in English were included. Two investigators independently performed study screening, data extraction, and quality control, with disagreements resolved by a third investigator. Visualization analyses were conducted using VOSviewer (v1.6.20), CiteSpace (v6.4.1), Bibliometrix (v5.4.1), and Scimago Graphica (v1.0.49).

**Results:**

222 publications (150 articles and 72 reviews) were included, involving 22 countries, 268 institutions, and 1,428 authors. China had the highest publication output, contributing 163 of the 222 included publications (73.42%), H-index (39), and the distribution of leading institutions and authors, but had a lower average citation count per item (ACI) than Australia (104.71) and Denmark (50.67). Zhejiang Chinese Medical University was the most productive institution (11 publications). Keyword co-occurrence, clustering, and burst analyses showed that metabolite-centered themes involving TMAO, SCFAs, IS, and pCS were prominent, while the temporal emergence of non-search-derived terms such as “tryptophan metabolism” and “AMPK signaling pathway” suggested increasing attention to upstream metabolic processes and downstream molecular mechanisms.

**Conclusion:**

Over the past five years, publication output in the field of DKD and gut microbial metabolites has increased substantially. Zhejiang Chinese Medical University in China demonstrated strong research productivity. Jin Juan had the highest publication count (six publications), closely followed by several authors with five publications. Frontiers in Pharmacology and Frontiers in Endocrinology were among the most frequent publication venues. Current research themes include associations between gut-derived uremic toxins and renal injury, the potential renoprotective roles of SCFAs, candidate metabolite biomarkers for DKD phenotyping and risk stratification, and interventions targeting microbial metabolic functions. Multicenter longitudinal studies and standardized clinical trials are needed to further validate the clinical relevance and translational potential of these research directions.

## 1 Introduction

Diabetic kidney disease (DKD) is one of the most common microvascular complications of diabetes and the leading cause of end-stage renal disease (ESRD) worldwide (Zhang Y. et al., 2025). According to the 11th edition of the International Diabetes Federation (IDF) Diabetes Atlas, approximately 589 million adults worldwide had diabetes in 2024, and this number is projected to increase to 853 million by 2050 (Genitsaridi et al., 2026). China has the largest population with diabetes worldwide, totaling 148 million; approximately one-third of these individuals also have chronic kidney disease (CKD) (Jia et al., 2025; Shang et al., 2026). Although conventional clinical management includes strict glycemic and blood pressure control and blockade of the renin-angiotensin system (RAS), a substantial proportion of patients still experience progressive loss of kidney function and ultimately develop irreversible kidney failure (Jia et al., 2025; Zali et al., 2025; Zhang W. et al., 2025). Elucidating novel mechanisms of DKD and identifying more effective and precise therapeutic targets have therefore become major priorities in endocrinology and metabolism.

In recent years, the gut-kidney axis has emerged as a potential framework for understanding interactions between the intestinal microbiota and kidney health in DKD (Rakshe and Gaikwad, 2026). Growing evidence suggests that gut microbiota-derived metabolites may act as potential mediators linking alterations in the intestinal microbial ecosystem with host metabolic, inflammatory, immune, and renal processes (Ni Y. H. et al., 2025; Ni Y. et al., 2025). For example, short-chain fatty acids (SCFAs) have been reported to activate specific receptors, attenuate inflammatory responses, and reduce oxidative stress, thereby potentially exerting renoprotective effects (Li et al., 2026; Lan et al., 2023). In contrast, gut-derived uremic toxins such as trimethylamine N-oxide (TMAO), indoxyl sulfate (IS), and p-cresyl sulfate (pCS) (Fang et al., 2021b; Shimizu et al., 2012; Watanabe et al., 2013) have been associated with renal tubular injury, oxidative stress, inflammation, and fibrotic responses in experimental studies (Fang et al., 2021a). However, the current evidence remains heterogeneous, ranging from observational associations to experimental mechanistic findings, and causal relationships have not been established for many gut microbiota-derived metabolites in DKD. Additionally, existing narrative, systematic, and mechanistic reviews primarily summarize biological findings and clinical implications, making them less effective for systematically charting the evolution of the field. In particular, they cannot readily provide an integrated quantitative assessment of how publication activity has evolved over time, how international and institutional collaborations have developed, which countries, institutions, and authors have been most influential, which research topics have gained or declined in prominence, and which emerging keywords and citation bursts signal rapidly developing areas of interest. These issues are important for understanding the intellectual and thematic evolution of research on gut microbiota-derived metabolites and DKD, but they remain insufficiently characterized.

Bibliometrics quantitatively evaluates publication characteristics within a research field using mathematical statistics and visualization techniques. By analyzing publication trends, country and institutional contributions, author networks, keyword co-occurrence, thematic clustering, and citation bursts, bibliometric analysis can quantitatively depict the knowledge structure, collaboration landscape, temporal evolution, and emerging frontiers of a research domain (Wei et al., 2022). Thus, bibliometric analysis can complement conventional evidence-based reviews by characterizing the structure and evolution of the research field itself, rather than primarily synthesizing the biological or clinical evidence within that field. Previous bibliometric research has examined global trends in diabetic nephropathy and intestinal flora, providing an overview of the research landscape, major contributors, collaboration patterns, and evolving topics in this broader field (Wang N. et al., 2025). However, this study focused on gut microbiota or intestinal flora as a whole and did not specifically examine gut microbiota-derived metabolites. Therefore, the development of metabolite-related research remains unclear. This includes research on specific metabolites, metabolomics, related signaling pathways, and metabolite-associated biomarkers. Because gut microbiota-derived metabolites may link microbial changes with host metabolic and renal responses, they warrant separate investigation. A metabolite-focused bibliometric analysis can therefore provide a more detailed view of research trends and changing priorities in this area.

Therefore, the present study conducted a multi-database bibliometric analysis of research on gut microbiota-derived metabolites and DKD published from 2014 to 2025. The study aimed to characterize publication trends, identify major contributing countries, institutions, and authors, assess collaboration patterns, examine the main research themes and their evolution, and identify emerging topics and research frontiers through keyword co-occurrence, clustering, and burst analyses. By addressing these aspects, this study provides a quantitative overview of the development and changing research priorities in gut microbiota-derived metabolite research in DKD.

## 2 Materials and methods

### 2.1 Data sources and search strategy

A systematic literature search was conducted on June 1, 2026, in four major bibliographic databases: Web of Science Core Collection (WoSCC), PubMed, Embase, and Scopus. WoSCC and Scopus were selected for their broad multidisciplinary coverage and detailed citation information, while PubMed and Embase provided complementary coverage of biomedical and clinical literature. These databases were searched to identify studies investigating the relationship between diabetic kidney disease (DKD), the gut microbiota, and gut microbiota-derived metabolites.

The search strategy was developed based on MeSH terms and related controlled vocabulary, supplemented by free-text synonyms and representative terms identified from relevant literature. The search terms were organized into three concept blocks: DKD-related terms, gut microbiota-related terms, and metabolite-related terms. For the metabolite concept, gut microbiota-derived metabolites were operationally defined as metabolites or molecular products whose production, transformation, or systemic availability depends substantially on gut microbial activity. This included metabolites directly produced by gut microbial metabolism, such as SCFAs, as well as co-metabolites generated through both microbial and host metabolism, such as TMAO, IS, and pCS. Secondary bile acids and other microbiota-associated metabolic products were also considered when their relationship with gut microbial metabolism and DKD was explicitly examined. To maximize retrieval sensitivity, the metabolite-related block included both broad terms, such as “metabolite,” “metabolomic,” and “lipidomic,” and specific metabolite names. Broad terms were used to capture studies based on untargeted or global metabolic profiling that did not identify specific metabolites in the title or abstract, whereas specific terms improved the retrieval of studies focusing on frequently investigated metabolites. The search strategy was adapted to the indexing systems and syntax of each database, and the complete search strategies are provided in Supplementary Table S1.

Because broad terms may also retrieve studies that are not directly relevant to the research question, all retrieved records were subsequently screened manually for relevance according to the predefined study scope. Studies were retained only when the investigated metabolites or metabolic profiles were sufficiently linked to gut microbiota-related metabolism and DKD. Thus, broad search terms were used to enhance sensitivity, whereas manual screening was used to improve specificity and ensure the relevance of the final literature set.

### 2.2 Inclusion and exclusion criteria

#### 2.2.1 Inclusion criteria

Studies were eligible if they met all of the following criteria: (i) Disease relevance: The study explicitly investigated DKD or diabetic renal involvement. This included human studies of patients with DKD, studies in which DKD was analyzed as a predefined subgroup or renal outcome of diabetes, and experimental studies using diabetic models with documented renal injury. (ii) Gut microbiota or metabolite relevance: The study investigated the gut microbiota, gut microbiota-associated metabolic alterations, or specific gut microbiota-derived metabolites. Gut microbiota-derived metabolites were defined according to the operational definition described in Section 2.1. (iii) Direct relationship between the two components: The study directly examined the relationship between DKD and the gut microbiota-derived metabolites. This relationship could be assessed through clinical or epidemiological associations, group comparisons, metabolomic or biomarker analyses, or mechanistic experiments. (iv) Experimental models: Animal and cell studies were eligible when the model represented diabetes-related renal injury or a DKD-relevant cellular process and the investigated gut metabolite component was directly related to the renal phenotype or mechanism. (v) Publication type: Original research articles and reviews were eligible. (vi) Publication characteristics: Articles published in English between database inception and December 31, 2025, were eligible.

#### 2.2.2 Exclusion criteria

The exclusion criteria were as follows: (i) Records identified from more than one database were excluded as duplicate publications. (ii) Conference abstracts, editorials, letters, commentaries, corrections, protocols, study registrations were excluded. (iii) Retracted articles and records accompanied by an expression of concern were excluded. (iv) Articles for which the relationship between DKD and gut microbiota-derived metabolites could not be adequately assessed because of insufficient information were excluded.

### 2.3 Literature screening and data collection

Two investigators (XY and XH) independently screened the retrieved records according to the prespecified eligibility criteria. After excluding commentaries, letters, books or book chapters, conference abstracts, retractions, errata, clinical trial protocols, and preprints, the eligible records were exported in plain-text format. Metadata retrieved from the four databases were then merged and imported into EndNote X9 for deduplication. Duplicate records were identified using available bibliographic identifiers, primarily DOI and PMID, together with combinations of title, first author, publication year, and journal information. When records from different databases contained incomplete or inconsistent metadata, potentially duplicate records were manually compared using the title, author list, publication year, journal, volume, pages or article number, and DOI where available. Records confirmed to represent the same publication were retained as a single entry, with the version containing the most complete bibliographic information preserved. Irrelevant records were subsequently excluded based on title and abstract screening, followed by full-text assessment when eligibility could not be determined with sufficient certainty. The two investigators independently assessed study eligibility, and disagreements were resolved through discussion, with unresolved cases adjudicated by a third senior investigator. A formal inter-reviewer agreement statistic, such as Cohen’s kappa, was not calculated during the original screening process.

For data collection, information from the final set of included publications was imported into Microsoft Excel 2021 to construct the bibliometric database. The collected information included publication title, authors, country or region, institution, journal, publication year, DOI, PMID where available, keywords, and citation information. Citation counts were obtained primarily from WoSCC. For publications not indexed in WoSCC, citation counts were manually retrieved from the corresponding journal websites when available. Citation-based indicators, including total citations, average citations per item (ACI), and H-index, were calculated using these harmonized citation data.

Quality control was performed throughout the screening and data collection processes to ensure the accuracy and consistency of the bibliometric database. The two investigators independently cross-checked the extracted bibliographic fields, including title, authors, publication year, journal, DOI, PMID where available, and citation information. Duplicate and retracted records were rechecked during database construction to ensure that they had been appropriately excluded. Inconsistencies in bibliographic metadata or citation information were verified against the original database records or corresponding journal webpages, and any remaining discrepancies were resolved through discussion between the two investigators or adjudication by a third investigator when necessary.

### 2.4 Visualization analysis

To enable multidimensional visualization and ensure reproducibility, we used multiple bibliometric tools with standardized operating parameters. GraphPad Prism 10 (Yue et al., 2025) was used to plot annual publication trends. VOSviewer 1.6.20 (van Eck and Waltman, 2010) and Scimago Graphica 1.0.49 (Hassan-Montero et al., 2022) were used jointly to construct co-occurrence network maps of countries, institutions, and authors, with minimum publication thresholds of 2, 1, and 1, respectively. The country threshold was set at two publications to reduce isolated single-publication nodes and improve the interpretability of the international collaboration network. Because research output at the institution and author levels was relatively dispersed, thresholds of one publication were retained to avoid excluding potentially relevant contributors. Full counting was used for the country, institution, and author analyses, whereby each publication contributed one full count to every participating country, institution, and author. CiteSpace 6.4.1 (Chen and Song, 2019) was used for keyword clustering and burst detection to trace the research frontier. The key CiteSpace parameters were as follows: time slicing from January 2014 to December 2025; 1 year per slice; g-index selection criterion (k = 25); and network pruning using Pathfinder and Pruning Sliced Networks to highlight the core structure. Finally, the Bibliometrix 5.4.1 R package (Aria and Cuccurullo, 2017) was used to support analyses of international collaboration patterns and leading journal characteristics.

## 3 Results

### 3.1 The trend of publication outputs

The literature search and screening process is shown in Figure 1. The initial search of the Web of Science, PubMed, Embase, and Scopus yielded 1,049 records. After restricting publication types, 281 conference abstracts, book chapters, letters, editorials, and other ineligible records were excluded, and 445 duplicates were removed. Screening of titles, abstracts, and full texts resulted in the exclusion of a further 101 publications that did not meet the eligibility criteria, including 61 unrelated to DKD and 40 unrelated to gut microbial metabolites. Ultimately, 222 eligible publications were included in the analysis, comprising 150 articles and 72 reviews. These publications were published from 2014 to 2025 and involved 268 institutions and 1,428 authors from 22 countries or regions.

**Figure 1:**
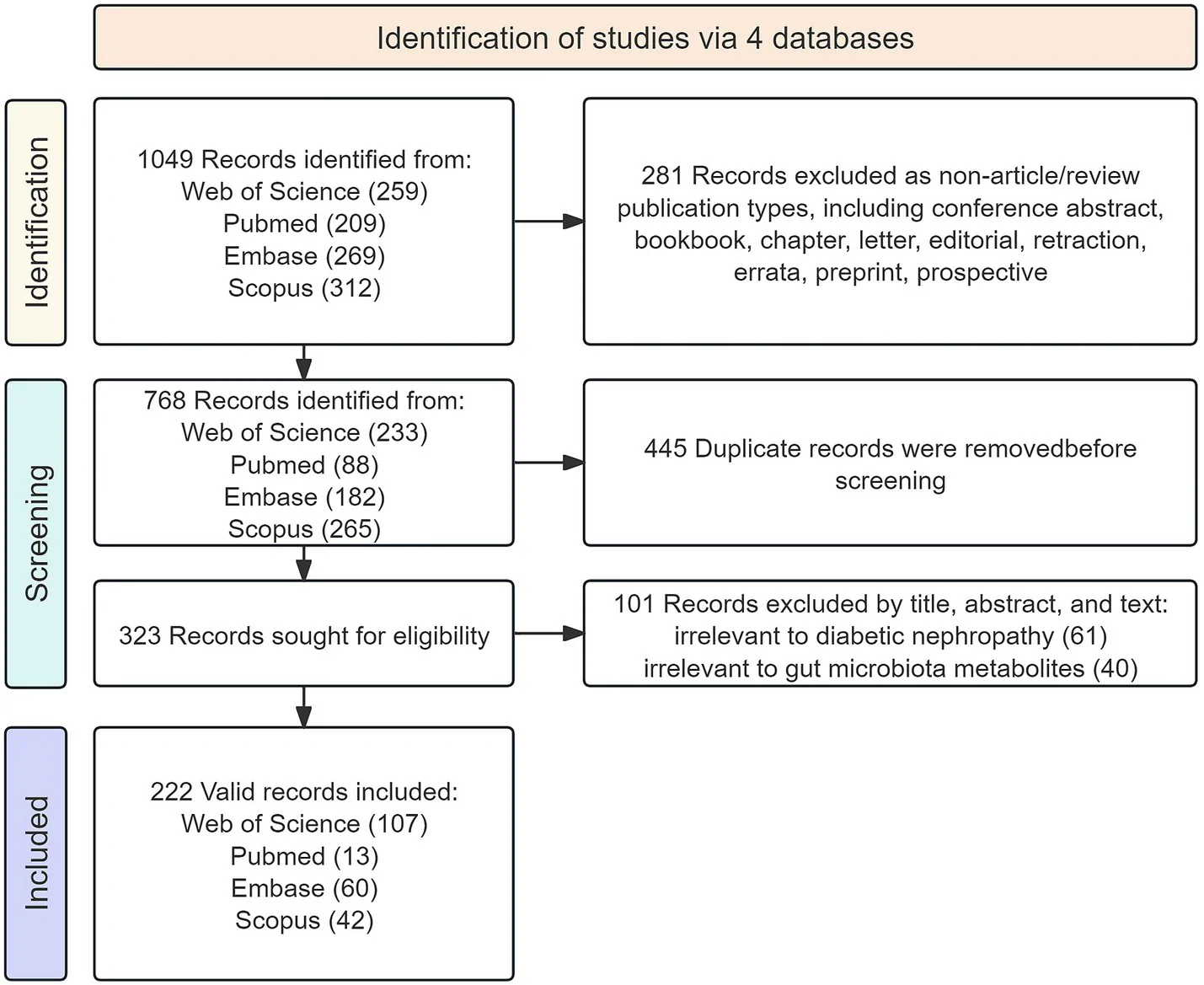
Flow diagram of the literature search and screening process.

Publication trend analysis showed a progressive increase in research activity in the field of DKD and gut microbial metabolites over the study period (Figure 2). From 2014 to 2018, the field was in its infancy, with no more than three publications per year. Research activity increased steadily from 2019 onward, followed by a more pronounced increase from 2022 to 2025, with 165 publications accounting for 74.32% of all included records. In 2025 alone, 61 publications were published, representing a 64.86% increase over the previous year. These findings demonstrate a substantial increase in publication activity in this research area in recent years. The observed increase may also partly reflect the general expansion of microbiome- and metabolomics-related research and changes in publication volume across biomedical journals.

**Figure 2:**
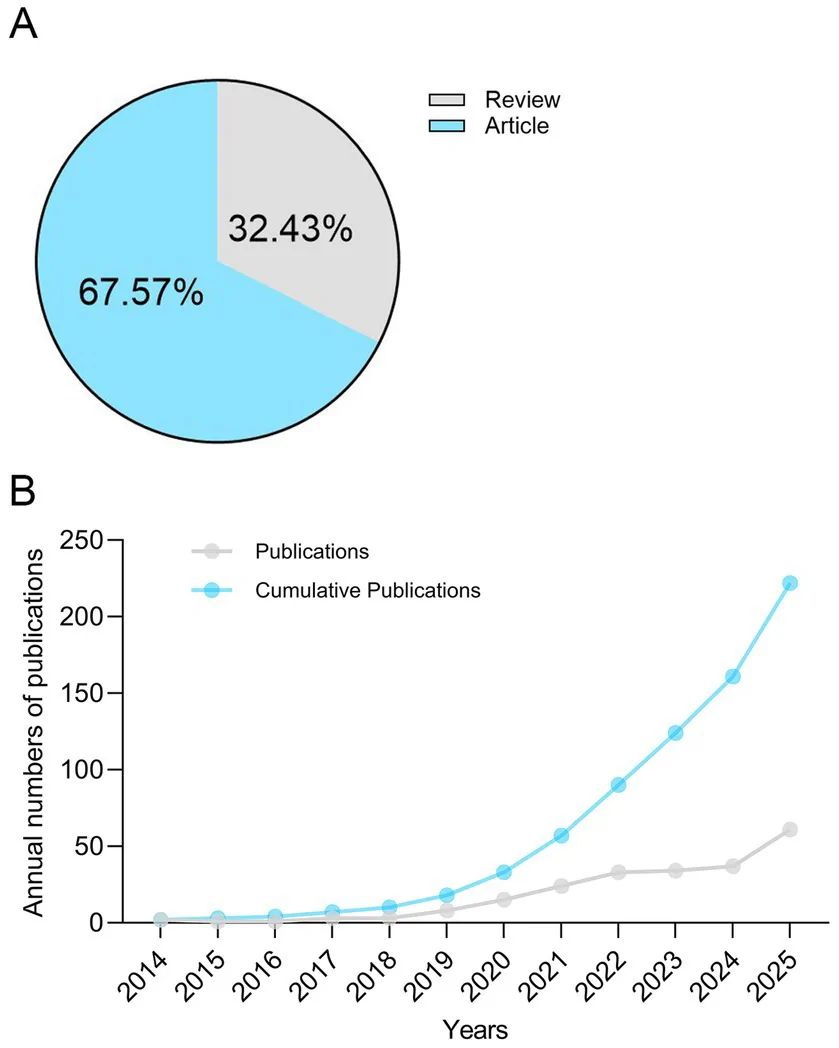
Trends of the annual publications related. **(A)** The pie chart shows the proportion of document types, where grey represents Reviews and blue represents Articles. **(B)** The line chart depicts the time-series evolution of annual publications, with the grey line representing the annual number of publications and the blue line representing the cumulative number of publications.

### 3.2 Distribution of subject categories

Dual-map overlay analysis of journals revealed citation trajectories and knowledge flows in this field (Figure 3A). In the map, black ellipses represent citing journals (the research frontier), whereas blue ellipses represent cited journals (the knowledge base); the horizontal and vertical axes of each ellipse reflect the number of authors and publications, respectively. Citing journals were concentrated primarily in the Molecular/Biology/Immunology and Medicine/Medical/Clinical fields, whereas cited journals were distributed mainly across Molecular/Biology/Genetics and Health/Nursing/Medicine. These citation trajectories indicate that pharmacological, clinical, and molecular biological research in this field is built on an interdisciplinary knowledge base centered on molecular genetics and basic medicine. Analysis of the subject categories represented by the included publications identified 34 disciplines (Figure 3B). Pharmacology & Pharmacy (46 publications, 20.72%) and Endocrinology & Metabolism (45 publications, 20.27%) predominated, followed by Biochemistry & Molecular Biology (24 publications, 10.81%) and Nutrition & Dietetics (22 publications, 9.91%). Thus, current research frequently focuses on pharmacological and nutritional modulation of endocrine and metabolic mechanisms.

**Figure 3:**
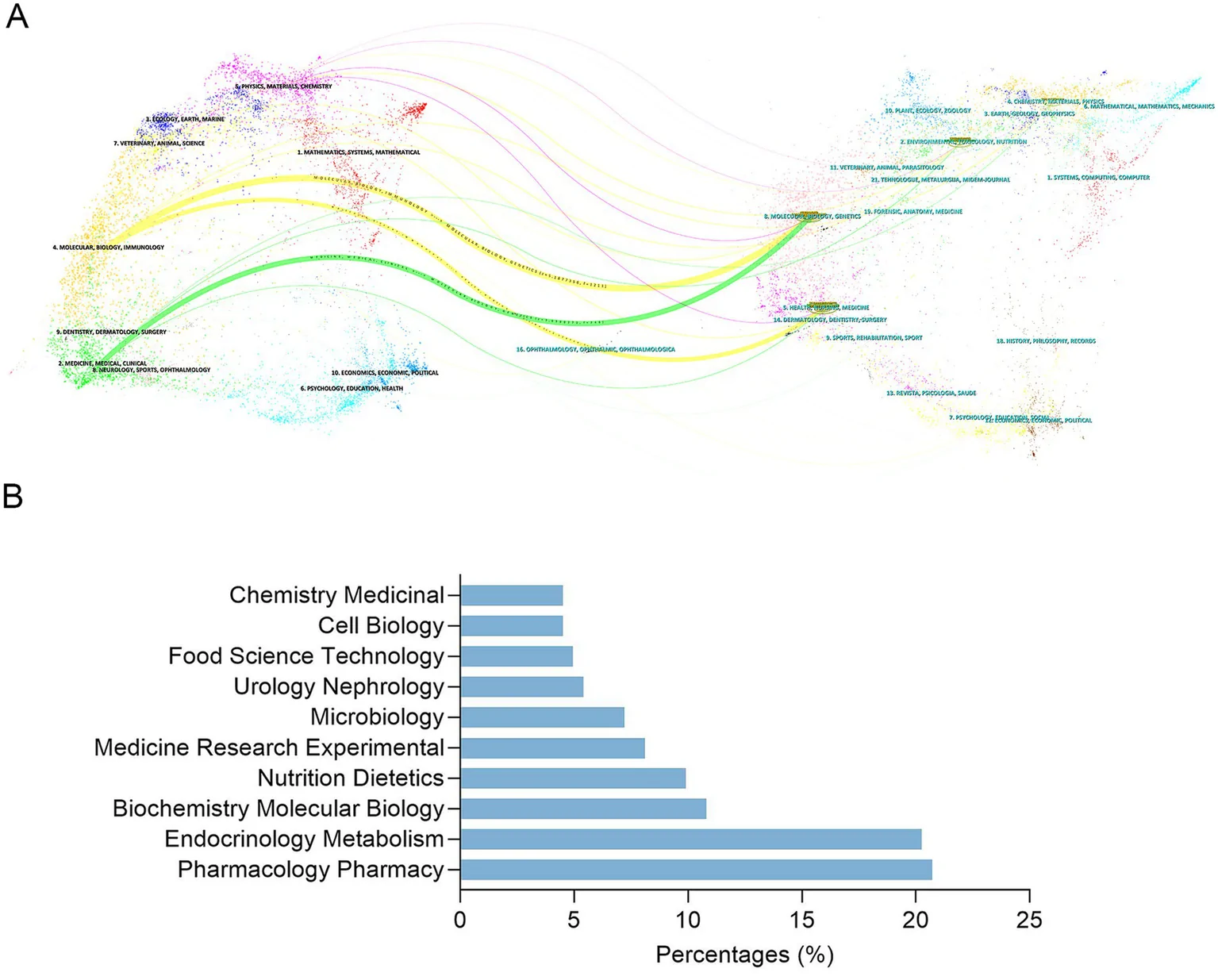
Dual-map overlay of journals and distribution of main subject categories. **(A)** The dual-map overlay of journal citations. The citing journals are on the left (representing research frontiers), and the cited journals are on the right (representing the knowledge base). Colored splines represent the citation trajectories, indicating the knowledge flow across different disciplines. **(B)** The bar chart shows the top 10 Web of Science subject categories by publication volume and their percentage share.

### 3.3 Analysis of countries/regions distribution

Research on DKD and gut microbial metabolites was conducted in 22 countries or regions worldwide. China ranked first in publication output (163 publications, 73.42%), followed by the United States (8 publications, 3.60%) (Table 1). Centrality quantifies how frequently a node lies along the shortest paths in a network and thereby reflects its topological importance; values greater than 0.1 define key nodes. China (0.33), Denmark (0.21), and the United States (0.10) had centrality values of at least 0.1, indicating core roles in the research network. The H-index is commonly used to assess academic influence. China ranked first with an H-index of 39, followed by the United States (H-index, 7), Australia (H-index, 6), and Romania (H-index, 6). However, China’s average citations per item (ACI; 27.22) remained substantially lower than those of Australia (104.71), Romania (88.50), and Denmark (50.67). This difference between publication output, H-index, and ACI suggests that country-level publication productivity and citation-based indicators did not show identical patterns. However, these citation-based comparisons should be interpreted cautiously because several countries contributed relatively few publications, and their ACI values may therefore be disproportionately influenced by one or a small number of highly cited papers. In addition, ACI is sensitive to publication age because earlier publications have had more time to accumulate citations. Therefore, these indicators should be considered complementary measures of publication impact rather than direct measures of research quality.

**Table 1:** Top 10 productive countries.

Rank	Country	Quantity	Proportion/%	Citations	ACI	H-index	Centrality
1	China	163	73.42	4,437	27.22	39	0.33
2	USA	8	3.60	158	19.75	7	0.10
3	Australia	7	3.15	733	104.71	6	0
4	Japan	7	3.15	329	47.00	4	0
5	Romania	6	2.70	531	88.50	6	0
6	India	5	2.25	34	6.80	2	0
7	Denmark	3	1.35	152	50.67	3	0.21
8	Poland	3	1.35	74	24.67	2	0
9	Korea	2	0.90	56	28.00	2	0.09
10	Malaysia	2	0.90	16	8.00	2	0

ACI, average citations per item.

The country or regional collaboration networks generated using Scimago Graphica 1.0.49 and Bibliometrix (Figures 4A,B) showed that collaboration was strongest between China and the United States, followed by China and the United Kingdom. Analysis of single-country publications (SCPs) and multiple-country publications (MCPs) (Figure 4C) further revealed marked differences in collaboration patterns. Although China had the largest overall output, most of its publications were SCPs, indicating a relatively low proportion of international collaboration. In contrast, all publications from Denmark involved international collaboration, whereas publications from Australia, Poland, and Malaysia were entirely SCPs. Overall, the geographic distribution of publications was highly concentrated, with substantial differences in international collaboration patterns across countries.

**Figure 4:**
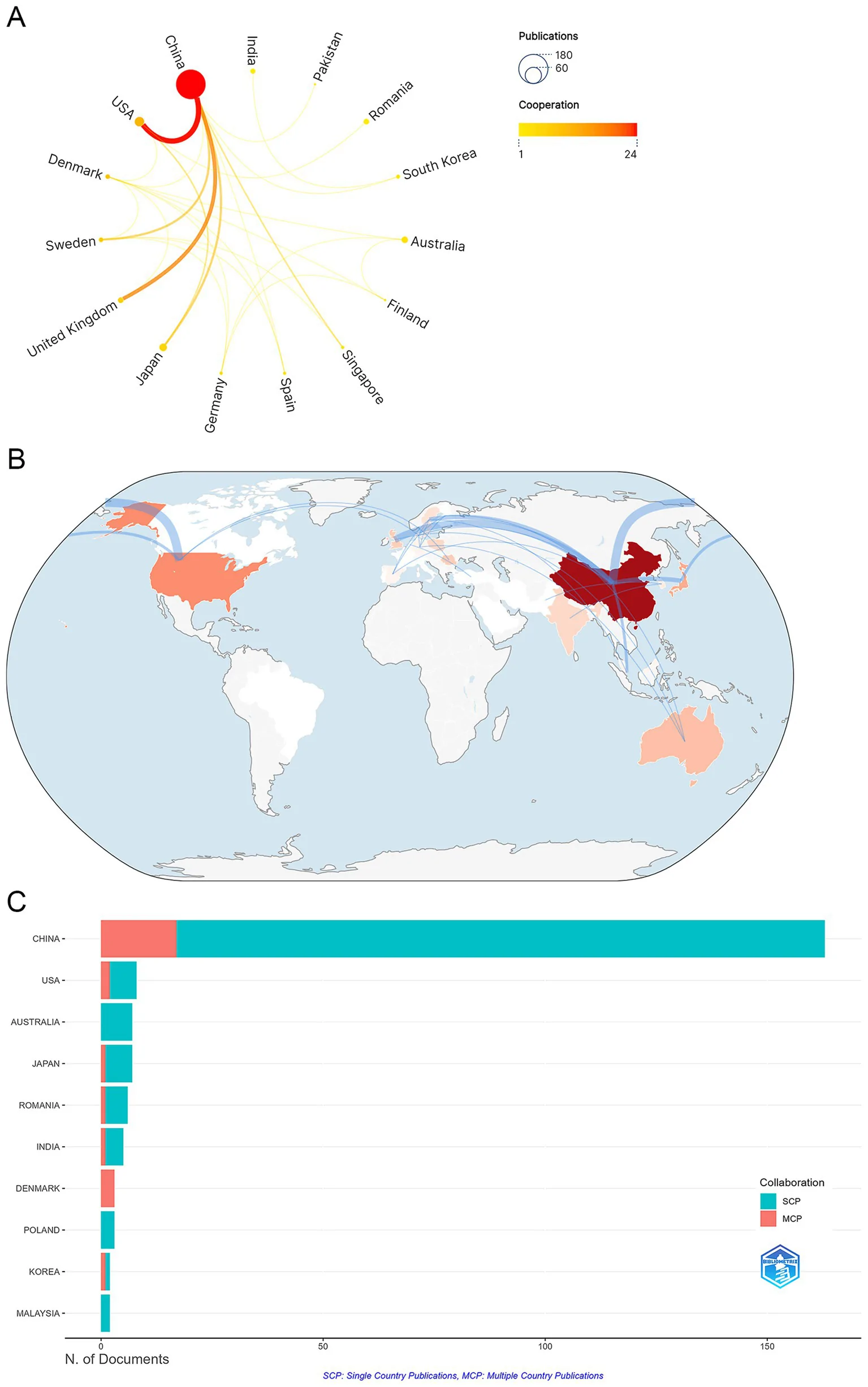
Global distribution of publications and international cooperation network of countries/regions. **(A)** Collaboration network map of countries/regions. The size of the node represents the total number of publications, and the color gradient of the lines (from yellow to red) represents the strength of cooperation. **(B)** Geographic distribution map of global publications. The color depth on the map reflects the number of publications, and the blue lines represent transnational academic collaboration paths. **(C)** The stacked bar chart statistics single/multiple country publications. Cyan represents Single Country Publications (SCP), and orange-red represents Multiple Country Publications (MCP).

### 3.4 Analysis of institutions distribution

A total of 268 institutions worldwide conducted research on DKD and gut microbial metabolites. Zhejiang Chinese Medical University ranked first with 11 publications, followed by Beijing University of Chinese Medicine and Zhengzhou University, each with 10 publications. All 10 most productive institutions were located in China (Table 2).

**Table 2:** Top 10 productive institutions.

Rank	Institution	Country	Quantity	Citations	ACI	H-index
1	Zhejiang Chinese Medical University	China	11	120	10.91	6
2	Beijing University of Chinese Medicine	China	10	197	19.70	8
3	Zhengzhou University	China	10	426	42.60	9
4	University of Chinese Academy of Sciences	China	9	162	18.00	7
5	Nanjing University of Chinese Medicine	China	8	126	15.75	4
6	China Pharmaceutical University	China	7	169	24.14	5
7	Sun Yat-sen University	China	7	151	21.57	5
8	China-Japan Friendship Hospital	China	6	174	29.00	4
9	Southeast University	China	6	315	52.50	5
10	Zhejiang University	China	6	110	18.33	4

ACI, average citations per item.

Institutional co-occurrence analysis illustrated collaboration networks among organizations (Figure 5A). Node size was positively correlated with publication output, while similar node colors and thicker connecting lines indicated closer collaboration. Among the 10 most productive institutions, collaboration was strongest between Zhejiang Chinese Medical University and Zhejiang University, followed by that between Beijing University of Chinese Medicine and China-Japan Friendship Hospital. In the temporal overlay map (Figure 5B), node color represents the mean publication year, ranging from dark blue for earlier activity to yellow-green for more recent activity. The dark-blue nodes for the University of Queensland and Princess Alexandra Hospital indicate that these institutions were active relatively early in the field. Conversely, the predominantly yellow-green nodes for leading institutions such as Zhejiang Chinese Medical University, Beijing University of Chinese Medicine, and Zhengzhou University indicate that their publication activity was concentrated in recent years and contributed substantially to the recent increase in publication output in this field.

**Figure 5:**
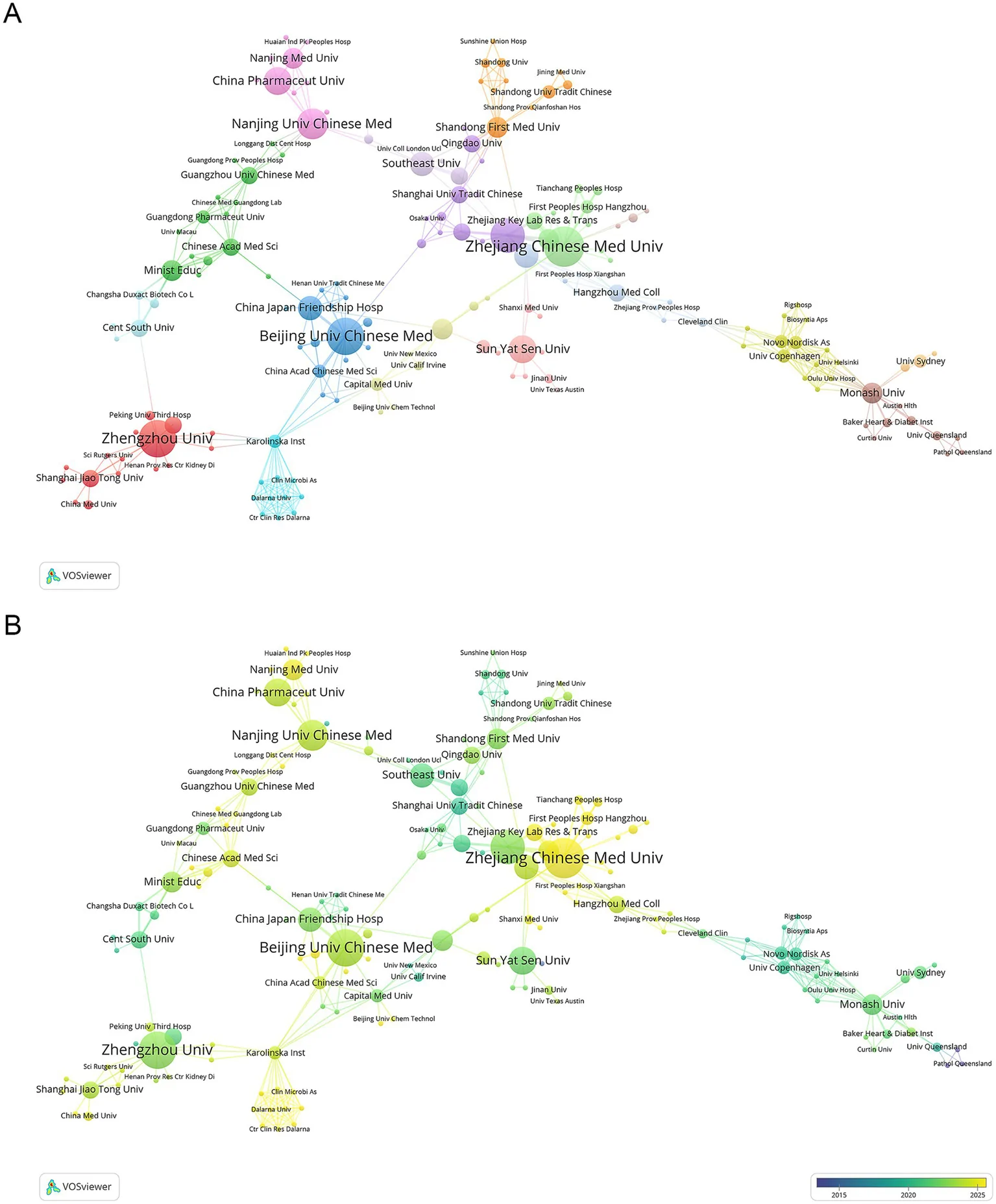
Co-occurrence network and overlay visualization of institutions. **(A)** Network visualization map of institutional collaboration. Node size represents the publication volume, node color indicates different collaborative clusters, and line thickness represents the strength of cooperation. **(B)** Overlay visualization map of institutions. Node color represents the average publication year, transitioning from dark blue for earlier average years to yellow-green for more recent average year.

### 3.5 Analysis of authors and authors collaborations

A total of 1,428 researchers worldwide contributed to studies on DKD and gut microbial metabolites, and all 10 most productive authors were from China. Jin Juan had the highest publication output, with six publications, while Ma Kunling, Ruan Xiongzhong, Li Ping, Shang Jin, Fu Zhengwei, He Qiang, Ni Yinhua, and Zheng Liujie each contributed five publications. These authors represented the main contributors in terms of publication productivity, although the difference in publication output between them was relatively small. In contrast, citation-based indicators showed a different pattern. Ma Kunling and Ruan Xiongzhong had the highest ACI and H-index (ACI, 62.60; H-index, 5), followed by Li Ping (ACI, 29.40; H-index, 4) and Cai Kedan (ACI, 24.75; H-index, 4). These findings indicate that publication productivity and citation impact varied across authors, with some authors showing relatively high citation-based impact despite having similar or lower publication output (Table 3).

**Table 3:** Top 10 authors.

Rank	Author	Country	Institution	Quantity	Citations	ACI	H-index
1	Jin Juan	China	Zhejiang Chinese Medical University	6	61	10.17	4
2	Ma Kunling	China	Southeast University	5	313	62.60	5
3	Ruan Xiongzhong	China	Southeast University	5	313	62.60	5
4	Li Ping	China	China-Japan Friendship Hospital	5	147	29.40	4
5	Shang Jin	China	Zhengzhou University	5	93	18.60	4
6	Fu Zhengwei	China	Zhejiang University	5	54	10.80	3
7	He Qiang	China	Zhejiang University	5	25	5.00	3
8	Ni Yinhua	China	Zhejiang University	5	54	10.80	3
9	Zheng Liujie	China	Zhejiang University	5	54	10.80	3
10	Cai Kedan	China	University of Chinese Academy of Sciences	4	99	24.75	4

ACI, average citations per item.

The author co-occurrence network revealed patterns of scholarly collaboration in this field (Figure 6A). Node size represents publication output, node color distinguishes collaboration clusters, and line thickness reflects the strength of collaboration. Li Ping, Cui Wen, Shang Jin, Wang Yaoxian, Zhang Yi, and Wang Tao emerged as core prolific authors. The overall network showed pronounced clustering: collaboration was close within individual teams, whereas links between teams were sparse, with some teams forming isolated subnetworks. Jin Juan, who had the highest publication count, was not included in the main co-occurrence cluster network. These findings indicate that research in this field is still conducted largely by small, independent teams and that cross-team and multicenter collaboration requires strengthening. In the temporal overlay map (Figure 6B), node color represents the mean publication year, from dark blue for earlier work to yellow-green for more recent work. Most core nodes were green to bright yellow, indicating that the contributions of prolific authors were highly concentrated within the past 5 years and that the relationship between DKD and gut microbial metabolites has recently become a research hotspot.

**Figure 6:**
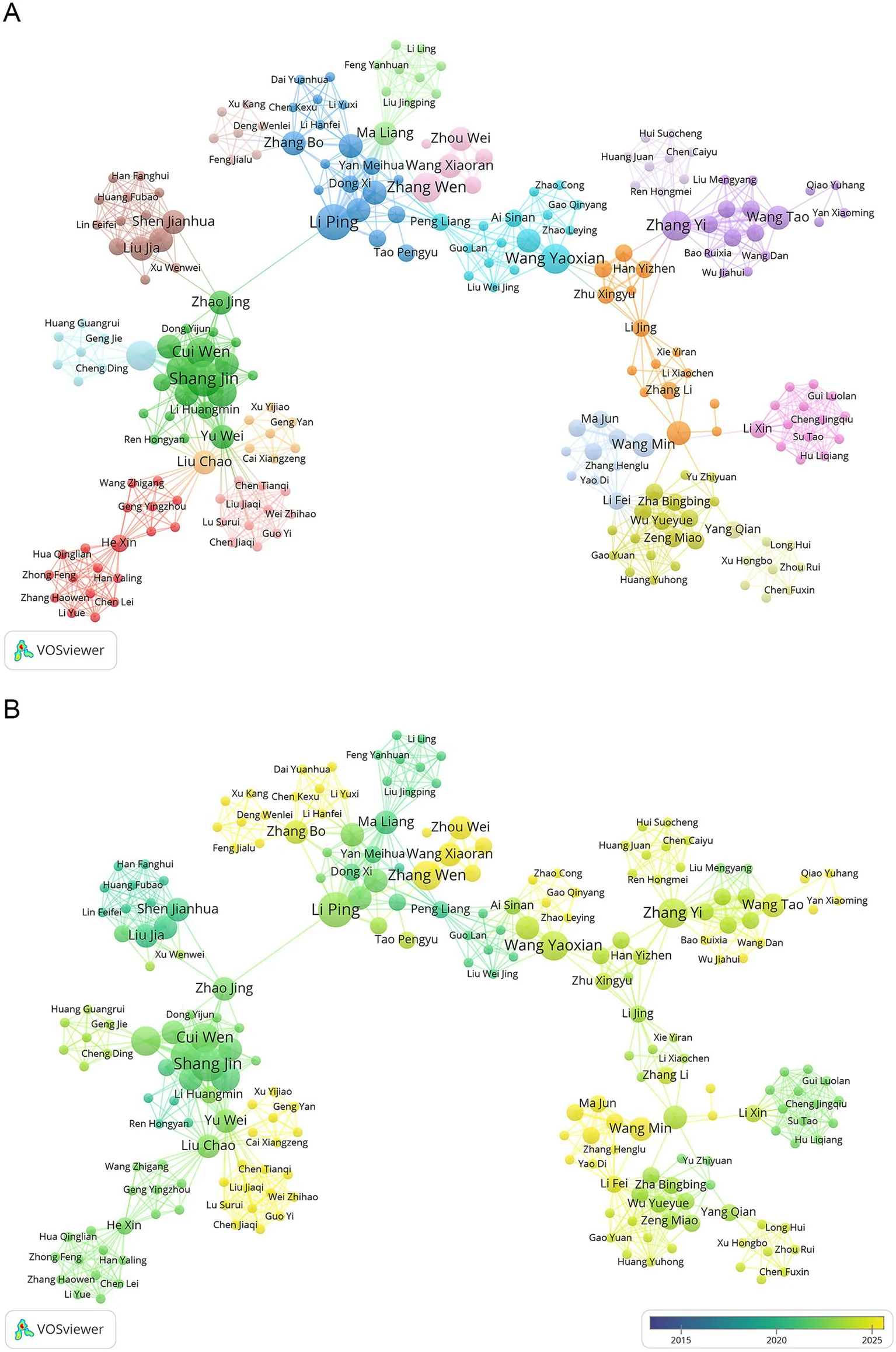
Co-occurrence network and overlay visualization of authors. **(A)** Network visualization map of author collaboration. Node size reflects the publication volume, node color distinguishes different core research teams (clusters), and line thickness represents the tightness of cooperation. **(B)** Overlay visualization map of authors. Node color indicates the average publication year of the authors’ achievements, with dark blue representing early researchers and yellow-green representing active scholars in recent years.

### 3.6 Analysis of journal distribution

The 222 included publications appeared in 121 academic journals. Eight of the 10 journals with the highest total citation counts were in Journal Citation Reports (JCR) Q1, indicating the high academic impact of research in this field (Table 4). Nutrients had the highest total citation count (10 publications; total citations [TC], 557), followed by Frontiers in Endocrinology (TC, 346) and Metabolism-Clinical and Experimental (TC, 301).

**Table 4:** Top 10 highly cited journals.

Rank	Journal	Quantity	Citations	ACI	H-index	IF (2025)	JCR
1	Nutrients	10	557	55.70	7	5.00	Q1
2	Frontiers in Endocrinology	15	346	23.07	10	4.60	Q2
3	Metabolism-Clinical and Experimental	1	301	301	1	11.90	Q1
4	Frontiers in Pharmacology	16	299	18.69	9	4.80	Q1
5	Journal of the American Society of Nephrology	1	295	295	1	9.40	Q1
6	Frontiers in Cellular and Infection Microbiology	4	283	70.75	3	4.80	Q1
7	Experimental and Clinical Endocrinology & Diabetes	2	251	125.5	2	1.70	Q4
8	Nature Communications	1	248	248	1	15.70	Q1
9	Biochimica et Biophysica Acta-Molecular Basis of Disease	2	206	103	2	4.20	Q1
10	Theranostics	3	200	66.67	3	13.30	Q1

ACI, average citations per item; IF, impact factor; JCR, journal citation reports.

Further analysis showed differences between publication volume and citation impact across journals. Frontiers in Endocrinology (15 publications; IF, 4.60; ACI, 23.07) and Frontiers in Pharmacology (16 publications; IF, 4.80; ACI, 18.69) were among the journals with the highest publication volumes in this field. In contrast, Metabolism-Clinical and Experimental (1 publication; IF, 11.90; ACI, 301), Nature Communications (1 publication; IF, 15.70; ACI, 248), and the Journal of the American Society of Nephrology (1 publication; IF, 9.40; ACI, 295) each contributed only one publication to the dataset. The high ACI values of these journals were therefore largely attributable to the citation performance of individual highly cited articles and did not reflect sustained publication activity within the present dataset. Overall, journal-level patterns were assessed by considering publication volume together with citation-based indicators, rather than ACI alone.

### 3.7 Analysis of hotspots and frontiers

CiteSpace 6.4.1 was used to analyze keywords in the included publications, and the 10 most frequent keywords are presented in Table 5. Several high-frequency terms, including “gut microbiota,” “diabetic nephropathy,” and specific metabolite-related terms such as “short-chain fatty acids,” were directly represented in the search strategy. Their high occurrence frequencies should therefore not be interpreted independently as evidence of research hotspots, because their prominence may partly reflect the design of the retrieval strategy. Accordingly, greater interpretive weight was placed on keywords that were not explicitly included in the search terms, particularly “inflammation” (57 occurrences) and “oxidative stress” (33 occurrences), which were less directly influenced by the predefined retrieval terms. Their high frequency indicates sustained scholarly attention to inflammatory and oxidative stress-related processes in research on gut microbial metabolites and DKD.

**Table 5:** Top 10 Keywords.

Rank	Keywords	Count	Centrality
1	gut microbiota	175	0.02
2	diabetic nephropathy	165	0.12
3	kidney disease	64	0.04
4	inflammation	57	0.04
5	short-chain fatty acids	56	0.07
6	chronic kidney disease	37	0.17
7	oxidative stress	33	0.05
8	gut microbial metabolites	26	0.03
9	metabolism	24	0.07
10	gut-kidney axis	23	0.04

In the keyword burst map (Figure 7A), blue lines indicate the overall period during which a keyword appeared, red lines indicate burst periods, and burst strength reflects the degree of concentrated scholarly attention. Early studies focused primarily on broad metabolic and pathological phenotypes: “diet induced obesity” appeared earliest, in 2014, and showed a particularly strong burst (3.35), while “oxidative stress,” which was not explicitly included in the retrieval terms, also exhibited an early burst. “Insulin resistance” (2.84) and “high glucose” (2.87) subsequently emerged as prominent topics. During the past 3 years, “tryptophan metabolism” (2.05) and “AMPK signaling pathway” (1.35), neither of which was directly specified in the search strategy, showed concentrated and ongoing bursts, indicating increasing scholarly attention to tryptophan metabolism and AMPK-related signaling in the recent literature.

**Figure 7:**
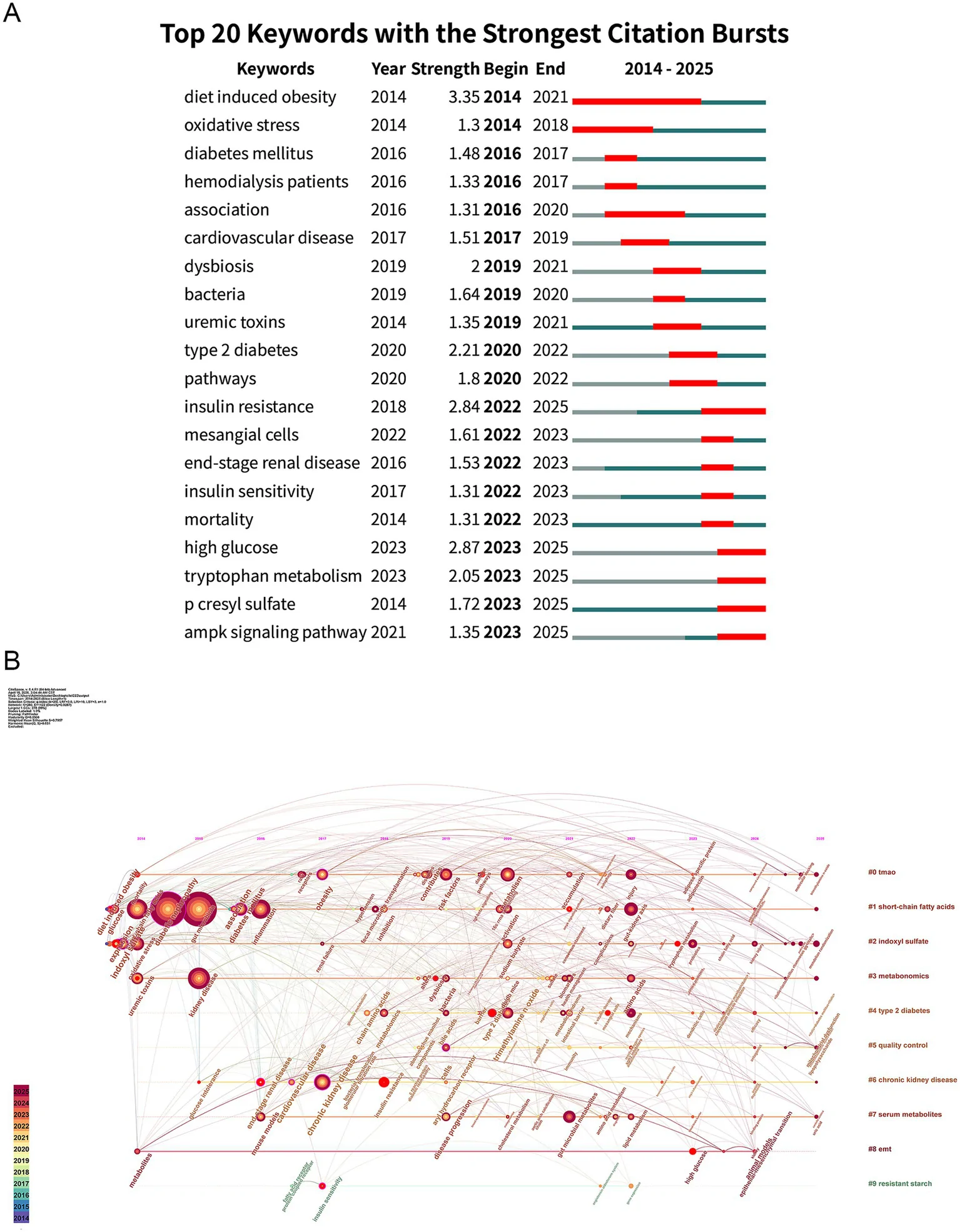
Burst detection of keywords and timeline cluster view. **(A)** Bar chart of the top 20 keywords with the strongest citation bursts. The blue line on the right represents the total time span of the keyword’s appearance, the red line represents the duration of its burst (explosive growth), and the “Strength” value represents the burst intensity. **(B)** Timeline view map of keyword clusters. The labeled text on the right represents different research cluster branches. The nodes on the horizontal solid lines indicate the year of the keyword’s first appearance, and the circles and lines reveal the evolution and interconnections of different cluster themes over time.

The timeline view (Figure 7B) depicts the knowledge structure and temporal evolution of keyword clusters. Labels on the right identify cluster themes, and nodes along the horizontal lines indicate the years in which publications first appeared. Several major metabolite-centered clusters were identified, including “#0 TMAO,” “#1 short-chain fatty acids,” and “#2 indoxyl sulfate.” These clusters demonstrate that specific microbial metabolites have constituted persistent thematic components of the literature. However, because TMAO, SCFAs, and indoxyl sulfate were explicitly represented in the search strategy, their cluster size and prominence may partly reflect retrieval design and should therefore be interpreted together with other keyword patterns rather than as independent measures of research prominence.

In summary, when considered alongside non-search-derived keywords and their temporal patterns, the results suggest that research attention has extended beyond individual metabolites toward inflammation, oxidative stress, tryptophan metabolism, and AMPK-related signaling. This pattern reflects changes in the topics receiving scholarly attention over time and provides a broader view of the thematic evolution of research on gut microbial metabolites and DKD.

### 3.8 Analysis of highly cited references and reference burst

Table 6 lists the 10 most frequently cited publications included in this study. The article by Yang et al. (2021), entitled Role of the gut microbiota in type 2 diabetes and related diseases, received the most citations (n = 301) and primarily reviewed the role of the gut microbiota in type 2 diabetes (T2D) and related diseases. The study by Li et al. (2020), Dietary fiber protects against diabetic nephropathy through short-chain fatty acid-mediated activation of G protein-coupled receptors GPR43 and GPR109A, ranked second with 295 citations and investigated the mechanism by which dietary fiber exerts renoprotective effects through SCFA-mediated activation of GPR43 and GPR109A. The review by Iatcu et al. (2021), Gut microbiota and complications of type-2 diabetes, ranked third with 291 citations and summarized associations between the gut microbiota and T2D-related complications. Collectively, the 10 most highly cited publications focused on associations of the gut microbiota with T2D and its complications; relationships of the gut microbiota with DKD and CKD progression; SCFAs and receptor-mediated signaling; and mechanisms involving gut-derived metabolites, uremic toxins, inflammation, and oxidative stress. Overall, these highly cited publications demonstrate that the field has expanded from microbial composition and disease associations toward gut microbial metabolites and their molecular mechanisms.

**Table 6:** Top 10 highly cited references.

Rank	Author	Title	Citations	Year	DOI
1	Ge Yang	Role of the gut microbiota in type 2 diabetes and related diseases	301	2021	10.1016/j.metabol.2021.154712
2	Yanjun Li	Dietary fiber protects against diabetic nephropathy through short-chain fatty acid-mediated activation of g protein-coupled receptors GPR43 and GPR109A	295	2020	10.1681/ASN.2019101029
3	Camelia Oana Iatcu	Gut microbiota and complications of type-2 diabetes	291	2021	10.3390/nu14010166
4	Zheng Zhou	Gut microbiota: an important player in type 2 diabetes mellitus	255	2022	10.3389/fcimb.2022.834485
5	Koichi Kikuchi	Gut microbiome-derived phenyl sulfate contributes to albuminuria in diabetic kidney disease	248	2019	10.1038/s41467-019-09735-4
6	Wei Huang	Short-chain fatty acids ameliorate diabetic nephropathy via GPR43-mediated inhibition of oxidative stress and NF-κB signaling	171	2020	10.1155/2020/4074832
7	Daniela Maria Tanase	Role of gut microbiota on onset and progression of microvascular complications of type 2 diabetes	158	2020	10.3390/nu12123719
8	Wei Huang	Short-chain fatty acids inhibit oxidative stress and inflammation in mesangial cells induced by high glucose and lipopolysaccharide	153	2017	10.1055/s-0042-121493
9	Megan Rossi	Protein-bound uremic toxins, inflammation and oxidative stress: A cross-sectional study in stage 3–4 chronic kidney disease	153	2014	10.1016/j.arcmed.2014.04.002
10	Alice Sabatino	Intestinal microbiota in type 2 diabetes and chronic kidney disease	152	2017	10.1007/s11892-017-0841-z

DOI, digital object identifier.

Reference burst detection facilitates the rapid identification of influential core publications and thereby reveals research frontiers and developmental trends. The study by Tao et al. (2019) showed the strongest citation burst (6.71), followed by that of Alicic et al. (2017) (burst strength, 5.21), indicating that these studies quickly attracted scholarly attention after publication. Among earlier high-burst publications, the study by Andrade-Oliveira et al. (2015) had a burst period from 2017 to 2020 and helped establish the field’s early research consensus. Notably, the aforementioned highly cited study by Kikuchi et al. (2019) not only accumulated many citations but also maintained a citation burst from 2020 through 2025, demonstrating the enduring influence of its mechanistic findings. In addition, the studies by Fang et al. (2021b) (burst strength, 3.89), Tuttle et al. (2022) (burst strength, 3.68), DeFronzo et al. (2021), and Zhang et al. (2022) collectively showed sustained, high-intensity bursts from 2023 to 2025. These citation bursts indicate that studies concerning novel mechanisms and therapeutic strategies related to gut-derived toxins have received increased scholarly attention in recent years, although burst activity alone does not establish their biological validity or predict their future scientific importance (Figure 8).

**Figure 8:**
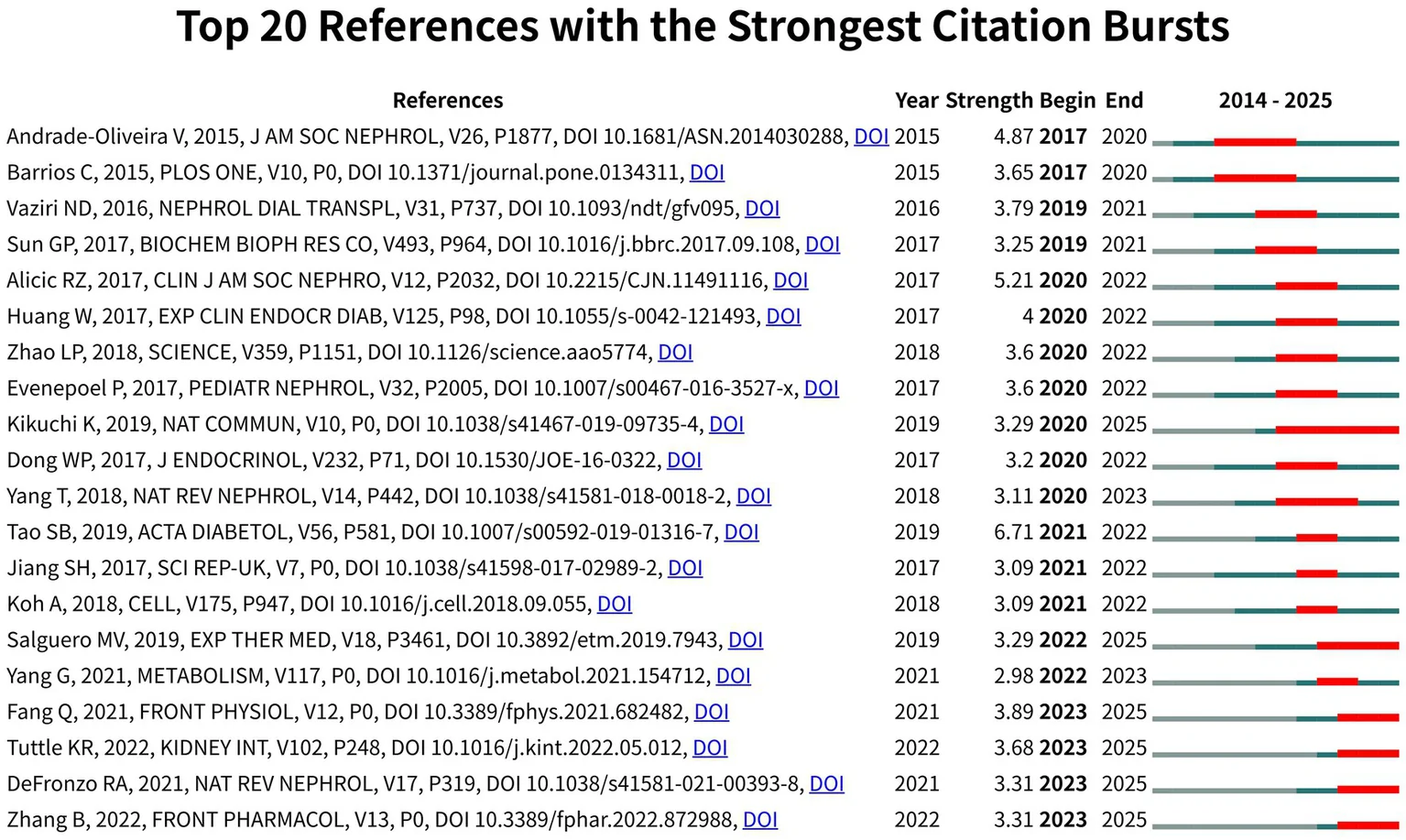
Top 20 references with the strongest citation bursts. This figure illustrates the burst dynamics of key literatures via a bar chart. The blue line on the right represents the overall time span of the reference’s citation, and the red line represents the concentrated burst period of high-frequency citations.

## 4 Discussion

### 4.1 General information

Through a multidatabase bibliometric analysis, this study characterized the development and thematic evolution of research on DKD and gut microbial metabolites from 2014 to 2025. Overall, publication activity increased substantially during the study period, particularly after 2022, indicating growing research activity in this area. This increase occurred alongside the broader expansion of microbiome- and metabolomics-related research. The geographic distribution of publications was highly concentrated, with China contributing the largest share of publications and several Chinese institutions emerging as major contributors. At the same time, international collaboration was evident, particularly between China and the United States and between China and the United Kingdom, although single-country publications remained common. This pattern highlights both the strong contribution of Chinese researchers to the literature and the continued development of international collaboration in this field. The keyword and clustering analyses further revealed a transition in research focus. Early studies were more closely associated with broad metabolic and pathological conditions, whereas later research increasingly addressed inflammatory and oxidative stress-related processes as well as specific metabolic and signaling pathways. Recent burst terms, including “tryptophan metabolism” and “AMPK signaling pathway,” reflect areas that have received increasing scholarly attention in recent years. Together, these findings suggest that research on gut microbial metabolites and DKD is becoming increasingly differentiated, with growing interest in metabolite-related mechanisms and their associated biological processes.

### 4.2 Hotspots and frontiers

The bibliometric analysis identified sustained scholarly attention to several gut microbiota-derived metabolites, including TMAO, IS, pCS, and SCFAs, as well as to inflammation, oxidative stress, tryptophan metabolism, and AMPK signaling pathway. These findings highlight four major areas of research that are discussed below: gut-derived uremic toxins and renal injury, SCFAs and their potential renoprotective effects, metabolomics-based candidate biomarkers and risk stratification in DKD, and gut microbiota-targeted interventions and prospects for clinical translation. The biological and clinical evidence discussed in these areas is derived from previous studies and is considered here to contextualize the research themes identified by the bibliometric analysis.

#### 4.2.1 Gut-derived uremic toxins and renal injury

The bibliometric analyses showed persistent metabolite-centered clusters involving and indoxyl sulfate, together with a recent keyword burst for p-cresyl sulfate. Because these metabolites were explicitly included in the search strategy, their prominence may partly reflect search-term selection and should therefore be interpreted with caution. More independently informative patterns were provided by the frequent occurrence and temporal evolution of “inflammation” and “oxidative stress,” which were not directly included in the search strategy. Their co-occurrence with uremic toxin-related themes suggests sustained scholarly attention to the relationships between gut-derived uremic toxins, inflammatory responses, oxidative stress, and renal injury in DKD. The biological mechanisms discussed below are based on previously published experimental and clinical studies and are used to contextualize these bibliometric patterns.

TMAO is generated through a two-stage metabolic process involving both the gut microbiota and the host. First, gut microorganisms convert dietary choline, phosphatidylcholine, and L-carnitine into trimethylamine (TMA). TMA is then absorbed from the intestine into the circulation and oxidized to TMAO, primarily by hepatic flavin-containing monooxygenase 3 (FMO3) (Wang et al., 2011; Koeth et al., 2013). In experimental models relevant to DKD, elevated TMAO levels have been associated with increased mitochondrial reactive oxygen species (mROS) production, activation of the NLRP3 inflammasome, and release of interleukin (IL)-1β and IL-18. NF-κB-related inflammatory signaling and TGF-β/Smad3 signaling may contribute to tubular injury and renal fibrosis (Fang et al., 2021b; Song et al., 2025; Yi et al., 2023). Experimental evidence also suggests that TMAO may be associated with inflammation and metabolic dysregulation through downregulation of vitamin D receptor (VDR) signaling (Zheng et al., 2024). These previously reported mechanistic findings provide biological context for the sustained prominence of TMAO-related themes in the temporal clustering identified in our bibliometric analysis. Nevertheless, the current evidence is derived primarily from basic and animal studies, and the causal role and clinical relevance of TMAO in patients with DKD require further validation.

IS is derived primarily from microbial metabolism of dietary tryptophan. Gut bacteria first convert tryptophan into indole, which is absorbed from the intestine, transported to the liver, and converted to IS through hydroxylation and sulfation (Lu et al., 2021). Because IS is highly protein-bound, its clearance depends substantially on tubular secretion; impaired kidney function therefore further promotes its accumulation (Sirich et al., 2014). Mechanistic studies, primarily in experimental models, have reported that IS can activate the aryl hydrocarbon receptor (AhR), increase reactive oxygen species production, and enhance Toll-like receptor 4 (TLR4)/JNK/NF-κB-related signaling. These changes have been associated with endothelial dysfunction, inflammatory mediator expression, tubular injury, and profibrotic responses (Zhao et al., 2020; Koppe et al., 2018; Zhao et al., 2019). The sustained keyword burst for “tryptophan metabolism” and the distinct temporal cluster for “indoxyl sulfate” suggest that research is expanding beyond the direct toxicity of IS to encompass its upstream microbial production and the mechanisms governing its renal clearance and systemic accumulation.

pCS is derived primarily from microbial fermentation of aromatic amino acids such as tyrosine and phenylalanine. Gut microorganisms first produce p-cresol, which is absorbed and transported to the liver, where it is converted mainly to pCS through sulfation (Gryp et al., 2017). Similar to IS, pCS is highly albumin-bound, which not only restricts renal clearance but also complicates interpretation of its circulating concentration and biological effects (Paroni et al., 2019). Experimental studies suggest that pCS may increase reactive oxygen species production and may contribute to vascular endothelial and tubular injury, together with activation of inflammatory and fibrotic signaling pathways. Suppressed renal AMPK activity and secondary activation of the NLRP3 inflammasome have also been implicated in pCS-associated kidney injury in experimental DKD models (Hong et al., 2023; Zhu et al., 2023). Although TMAO, IS, and pCS differ in their origins and upstream pathways, their reported biological effects converge on pathways involving inflammation, oxidative stress, and structural kidney injury. TMAO is more closely associated with mROS/NLRP3 and TGF-β/Smad signaling, IS prominently involves AhR-related pathways, and pCS is characterized more by oxidative and metabolic stress associated with AMPK dysregulation (Fang et al., 2021a). The temporal patterns of the TMAO- and indoxyl sulfate-related clusters, together with the recent keyword bursts for “p-cresyl sulfate” and “tryptophan metabolism,” further suggest sustained scholarly attention to the associations between gut-derived uremic toxins and renal injury in DKD.

#### 4.2.2 SCFAs and potential renoprotective effects

SCFAs formed a prominent and persistent metabolite-centered theme in the keyword and timeline analyses. Because SCFA-related terms were explicitly included in the retrieval strategy, their high frequency and cluster prominence may partly reflect search-term selection and should not be interpreted independently as evidence of research importance. More informative evidence of thematic development was provided by the association of the SCFA cluster with non-search-derived terms such as “inflammation,” “oxidative stress,” and the recent burst of “AMPK signaling pathway.” Together, these patterns suggest increasing scholarly attention to the potential links between the dietary fiber-gut microbiota-SCFA axis and inflammatory, oxidative, and metabolic signaling mechanisms in DKD.

SCFAs, primarily acetate, propionate, and butyrate, are produced through colonic microbial fermentation of nondigestible dietary carbohydrates (Koh et al., 2016). In addition to serving as important metabolic substrates, SCFAs help maintain intestinal epithelial barrier integrity, regulate immune and inflammatory responses, and influence energy metabolism and glucose homeostasis (Ma et al., 2022). A highly cited study included in our analysis showed that dietary fiber attenuated diabetic kidney injury by increasing SCFA production and activating GPR43 and GPR109A signaling (Li et al., 2020). Reduced fecal SCFA concentrations or a lower abundance of SCFA-producing bacteria have also been observed in populations with diabetes and kidney disease (Winther et al., 2020; Zhong et al., 2021). A two-cohort study further found that higher dietary butyrate intake and circulating butyrate concentrations were each independently associated with better kidney function in adults with diabetes (Zhao et al., 2025). These previously published findings provide clinical and biological context for the prominence of SCFA-related themes in our bibliometric results. However, most available studies are cross-sectional or observational and cannot establish that increasing circulating SCFA concentrations directly improves estimated glomerular filtration rate (eGFR) or reduces albuminuria.

Experimental studies suggest that activation of GPR43 and GPR109A may partly explain the potential anti-inflammatory renal effects associated with SCFAs (Li et al., 2020). Signaling through these receptors has been reported to attenuate proinflammatory and profibrotic responses in experimental models. GPR43-related signaling has also been reported to attenuate oxidative stress and NF-κB activation in experimental studies (Li et al., 2023; Huang et al., 2020). SCFAs may additionally enhance intestinal epithelial barrier function and reduce the translocation of lipopolysaccharide (LPS) and the resulting systemic inflammatory burden (Peng et al., 2009; Feng et al., 2018). Recent studies have extended the mechanisms of SCFA action to metabolic and epigenetic regulation, including preservation of mitochondrial homeostasis through the AMPK/SIRT1/PGC-1α pathway and regulation of autophagy through mechanisms involving histone deacetylase 2 (HDAC2)/unc-51-like autophagy-activating kinase 1 (ULK1) (Yu et al., 2023; Ye et al., 2024; Ma and Wang, 2022; Du et al., 2020). Thus, the continued activity of the “short-chain fatty acids” cluster and the recent burst of “AMPK signaling pathway” suggest increasing scholarly attention to microbial metabolic functions and their potential effects on the host, beyond descriptive analyses of microbial composition.

#### 4.2.3 Metabolomics-based candidate biomarkers and risk stratification in DKD

The bibliometric analysis identified increasing scholarly attention to metabolomics and specific microbial metabolites, whereas their potential diagnostic and prognostic roles are based on previously published clinical studies rather than validation performed in the present analysis. Keyword analysis identified themes including “metabolism,” “gut microbial metabolites,” “metabolomics,” and “serum metabolites.” The recent sustained activity of “tryptophan metabolism” and “p-cresyl sulfate” suggests increasing scholarly attention to the identification and evaluation of specific metabolites. Current evidence supports their consideration primarily as candidate biomarkers or risk-stratification markers rather than as established early diagnostic biomarkers. The clinical evidence summarized below is derived from previously published studies and is presented to contextualize this bibliometric pattern. More broadly, metabolomics has been increasingly applied to characterize metabolic alterations and identify candidate biomarkers in diabetes and DKD, although their clinical utility requires further validation in well-designed prospective studies (Pandey, 2024).

Previous clinical studies have suggested that TMAO may have potential value as a candidate biomarker. Studies in patients with T2D have reported higher circulating TMAO concentrations in those with DKD or microalbuminuria, together with associations between TMAO-related metabolism and renal involvement (Yang et al., 2022; Huo et al., 2023). In a prospective cohort of individuals with type 1 diabetes, higher plasma TMAO concentrations were associated with all-cause mortality, cardiovascular events, and adverse renal outcomes (Winther et al., 2019). However, these associations became nonsignificant after further adjustment for baseline eGFR, suggesting that circulating TMAO may partly reflect impaired kidney function rather than serving as an independent disease-specific biomarker (Winther et al., 2019). TMAO concentrations are also influenced by dietary patterns, gut microbial composition, medication exposure, and renal clearance. This complexity limits its disease specificity and currently precludes its use as an established standalone biomarker for early DKD diagnosis.

IS and pCS are likewise more appropriately regarded as candidate biomarkers than as established diagnostic markers. These observations are based on previously published clinical and metabolomic studies rather than validation performed in the present bibliometric analysis. Targeted metabolomics studies in relatively small T2D populations have found that several gut microbiota-related metabolites, including IS and pCS, are altered during early DKD and are associated with markers of podocyte, proximal tubular, and vascular endothelial injury (Winther et al., 2020; Balint et al., 2023c; Balint et al., 2023a; Balint et al., 2023b). Studies of type 1 diabetes stratified by albuminuria have also identified significant differences in several microbial metabolites, including IS (Winther et al., 2020). These previously reported findings suggest that measuring IS and pCS in plasma or urine may facilitate DKD phenotyping and risk stratification and may, in particular, help compensate for the limited ability of albuminuria to reflect tubular injury. However, both IS and pCS accumulate as GFR declines and are highly protein-bound; their concentrations are also influenced by diet, intestinal transport, medication exposure, and specimen type. Existing studies are further limited by small sample sizes, substantial population heterogeneity, and a lack of external validation. Future longitudinal, multicenter, and geographically diverse cohorts should standardize sample collection, preprocessing, and analytical procedures and include independent external validation. Metagenomic, metabolomic, and clinical variables could then be integrated into multimetabolite prediction models, with particular emphasis on their incremental predictive value beyond conventional measures such as eGFR and UACR. Taken together, the bibliometric results indicate growing research attention to metabolomics and specific microbial metabolites, whereas their diagnostic and prognostic utility remains to be established through independent, prospective clinical validation.

#### 4.2.4 Gut microbiota-targeted interventions and prospects for clinical translation

Temporal and burst analyses indicated increasing scholarly attention to microbiota-targeted interventions, while the therapeutic mechanisms and effects discussed below are derived from existing experimental and clinical literature. Temporal keyword evolution and reference burst analyses showed sustained interest in metabolic targets such as “p-cresyl sulfate,” “tryptophan metabolism,” “short-chain fatty acids,” and “AMPK signaling pathway.” These bibliometric signals suggest increasing scholarly attention to interventions targeting microbial metabolic functions and their potential clinical translation, rather than demonstrating that such strategies are already clinically established. The intervention evidence discussed below is derived from previously published experimental and clinical studies and is used to contextualize this evolving research theme.

The first major intervention strategy is to reduce the production, absorption, and downstream effects of harmful gut-derived metabolites. Previous studies suggest that, provided nutritional adequacy is maintained, increasing fermentable carbohydrate intake and avoiding excessive protein intake may help prevent colonic fermentation from shifting toward the production of IS and pCS precursors. Broader dietary modification may also reduce exposure to TMA precursors and endotoxins. Published studies suggest that probiotics, prebiotics, and synbiotics may provide adjunctive benefits by remodeling microbial function and improving intestinal barrier integrity, but their efficacy is influenced by strain composition, dose, intervention duration, and the target population and therefore requires confirmation in high-quality studies (Wang X. et al., 2025). In experimental DKD studies, some traditional Chinese medicine formulations and natural products reduced circulating IS, pCS, TMAO, or LPS concentrations while modulating toxin-producing bacteria, AhR/TLR4 signaling, and inflammation-related pathways (Yi et al., 2023; Zhao et al., 2020; Hong et al., 2023; Dong et al., 2023; Gao et al., 2021). Vitamin D may also regulate the gut microbiota-TMAO-VDR axis (Zheng et al., 2024), while dapagliflozin has shown potential effects related to modulation of the gut-kidney axis in experimental studies (Ni Y. H. et al., 2025). However, although sodium-glucose cotransporter 2 (SGLT2) inhibitors have established clinical kidney benefits, direct evidence remains insufficient to determine the extent to which these benefits are mediated by changes in the gut microbiota and its metabolites (DeFronzo et al., 2021).

The second major intervention strategy is to promote the production and restore the functions of beneficial gut-derived metabolites. Dietary fiber and prebiotics provide fermentable substrates for SCFA-producing bacteria. Under appropriate strain and ecological conditions, probiotics or synbiotics may also promote SCFA production by remodeling microbial composition and function. Previously published experimental evidence showing that dietary fiber can activate GPR43 and GPR109A provides biological support for this intervention pathway, although its clinical effectiveness remains to be established (Li et al., 2020). Experimental studies have reported that direct supplementation with butyrate or other SCFAs can attenuate inflammation, metabolic dysregulation, and kidney injury in experimental models through GPR43/GPR109A-dependent signaling, the AMPK/SIRT1/PGC-1α pathway, and HDAC2/ULK1-related epigenetic and autophagic regulation (Li et al., 2023; Ma and Wang, 2022; Du et al., 2020). Importantly, direct SCFA supplementation and restoration of SCFA-producing microbial communities are distinct intervention strategies. They may differ in luminal and systemic exposure, the durability of restored microbial function, and renal safety, and their effects may vary according to CKD stage and the host microbial ecosystem.

Clinical translation must also account for interindividual microbial heterogeneity, geographic and dietary differences, concomitant medications, kidney function, and the context-dependent production of metabolites. Most current intervention evidence is derived from animal experiments or small clinical studies with short follow-up, and intervention formulations, doses, and regimens vary substantially among studies. Moreover, whether interventions consistently modulate their intended microbial metabolic targets has not been adequately validated, and evidence concerning long-term kidney outcomes remains limited. Future prospective clinical trials with adequate sample sizes should evaluate standardized intervention regimens, dynamically validate targeted mechanisms by integrating metagenomics and metabolomics, prespecify clinically meaningful kidney outcomes, and perform independent external validation across geographic regions and populations. In light of the temporal trends identified in our bibliometric analysis and the biological evidence reported in previous studies, future research may increasingly focus on the precise modulation of microbial metabolic functions and key metabolites rather than microbial composition alone. However, whether such strategies translate into clinically meaningful kidney benefits requires confirmation in adequately powered prospective trials.

### 4.3 Limitations and prospects

Although this study used systematic bibliometric methods to provide a relatively comprehensive overview of research trends and frontiers concerning DKD and gut microbial metabolites, several limitations should be acknowledged (Zhang Y. et al., 2025). Although data were obtained from four major databases—the Web of Science, PubMed, Embase, and Scopus—only English-language original articles and reviews were included to ensure data quality and compatibility with visualization software. Important studies published in other languages or in journals not indexed by these databases may therefore have been missed (Genitsaridi et al., 2026). Bibliometric analysis is subject to a time-lag effect. Although publication output increased rapidly in 2025, citations and burst strength require time to accumulate; consequently, some recently published studies with potentially high impact may not yet be fully represented in the current knowledge map (Jia et al., 2025). Although keywords were standardized, authors may use different terms for specific metabolites, signaling pathways, and related concepts, which may reduce the precision of keyword clustering and hotspot identification. In addition, keyword co-occurrence, clustering, and burst analyses may be influenced by search-term selection (Shang et al., 2026). Citation-based indicators are influenced by publication age, database coverage, and publication volume. ACI values for countries with few publications may be disproportionately affected by highly cited papers. In addition, the lower ACI observed for China may partly reflect its more recent increase in publication activity and shorter citation-accumulation time, rather than lower research quality or scientific influence.

Future research should advance mechanistic investigation and clinical translation on the basis of current hotspots and frontiers. First, metagenomics, metabolomics, and other multi-omics approaches should be integrated to elucidate how TMAO, IS, pCS, SCFAs, and newly identified microbial metabolites contribute to inflammation, oxidative stress, and renal fibrosis in DKD. Second, large, multicenter, prospective cohorts are needed to validate candidate metabolites for the early diagnosis, risk stratification, and prognostic assessment of DKD and to develop integrated models combining clinical measures with multi-omics features. In addition, high-quality randomized controlled trials should evaluate the long-term efficacy and safety of dietary modification, probiotics or prebiotics, and interventions targeting metabolic pathways. Such trials should account for heterogeneity in microbial composition, metabolic phenotype, and concomitant medications, explore precision stratification and individualized interventions, and facilitate the translation of gut microbial metabolite research from mechanistic studies to clinical practice.

## 5 Conclusion

This study systematically mapped the research landscape and developmental trends of DKD and gut microbial metabolites from 2014 to 2025. Publication activity increased substantially in recent years, with China contributing the largest share of publications and several Chinese institutions emerging as major contributors. Collaboration analyses revealed marked national and institutional clustering. Keyword co-occurrence, temporal clustering, and burst analyses identified several dominant research themes, including research on gut-derived uremic toxins and their associations with renal injury, investigation of the potential renoprotective roles of SCFAs, evaluation of gut microbial metabolites as candidate biomarkers for DKD phenotyping and risk stratification, and exploration of interventions targeting microbial metabolic functions. These themes reflect patterns of research activity and scholarly attention identified in the published literature rather than biological mechanisms or clinical effects independently established by the present bibliometric analysis. More broadly, publication and citation patterns should not be interpreted as direct indicators of the strength, quality, or causal validity of the underlying evidence. Future studies should strengthen international collaboration and validate candidate metabolites and intervention strategies through prospective, multicenter, and standardized clinical research.

## Data Availability

The original contributions presented in the study are included in the article/Supplementary material, further inquiries can be directed to the corresponding authors.
